# Mini-Incision Living Donors Nephrectomy Using Anterior Muscle-Splitting Approach with Hybrid Technique

**Published:** 2010-02-01

**Authors:** N. Nezakatgoo, M. M. Hashad, A. Saharia, L. W. Moore, A. Osama Gaber

**Affiliations:** 1 *Transplantation Division, University of Tennessee, Memphis, TN, USA,*; 2 *Transplant Institute, Memphis, TN, USA, *; 3 *Department of Urology, University of Alexandria, Alexandria, Egypt,*; 4 *The Methodist Hospital Transplant Center, Houston, TX, USA*

**Keywords:** Transplant, kidney, laparotomy, laparoscopy, nephrectomy

## Abstract

Background: Significant morbidity is associated with standard open flank living donor nephrectomy. Laparoscopic donor nephrectomy is criticized for a steep learning curve and a tendency to avoid the right kidney. The anterior muscle-splitting technique uses principles or advantages of an open extraperitoneal approach with minimal morbidity and the advantageous muscle-splitting (instead of cutting) procedure.

Objective: To compare mini-incision laparoscopic instrument-assisted (MILIA) live donor nephrectomy using a muscle-splitting technique to the standard open-flank donor nephrectomy (ODN) approach for efficacy and safety.

Methods: MILIA living donor nephrectomies were performed in 119 donors and compared to a cohort of open-flank nephrectomy donors (n=38) from the same center. Both donor groups were matched for body mass index as well as other personal characteristics.

Results: The mean donor age was 35 (range: 18–60) years. The right kidney was procured in 28% of cases. The majority of donors were female (58%) and Caucasian (60%). No differences were observed between MILIA and ODN donors for the age, gender and ethnicity. However, MILIA donors experienced a longer mean±SD operative time (234±47 vs. 197±33 min, p<0.0001) but a shorter hospital stay (4±1 vs. 6±3 days for the ODN group, p<0.0001) and less intraoperative blood loss (215±180 vs. 331±397 mL, p<0.02). No difference was found in the number of units of blood transfused (0.13±0.6 vs. 0.34±1.0 units, p=0.13). Right-sided kidneys were almost equally harvested in both groups (29% of MILIA donors vs. 26% of ODN donors). Post-operatively, MILIA donors had a significantly lower mean pain scores at one week and one month after surgery (p<0.001). They showed significant better post-operative recovery—earlier stopping of pain medications and restoration of other preoperative activities. Moreover, they were better satisfied with their scar appearance. Scores on the short form-36 quality of life questionnaire were comparable for both groups.

Conclusion: MILIA is a viable option as an alternative for pure laparoscopic donor nephrectomy. MILIA appears to be as safe as open donor nephrectomy and may provide advantages over ODN, such as smaller incision, shorter hospital stay, and less incisional pain. Patient recovery and satisfaction after MILIA are excellent. This technique avoids the possibility of adhesive intestinal obstruction and also improves handling of major complications (e.g., bleeding) of laparoscopic donor nephrectomy. Utilization of this hybrid technique is particularly feasible on smaller (BMI<24 kg/m^2^) and medium-sized (BMI<28 kg/m^2^) donors. We believe that this technique should be adopted by centers that have limited advanced laparoscopic surgical experience and also it could be used selectively for the right donor nephrectomies, even in centers performing hand assisted donor nephrectomies by including a small patch of inferior vena cava for a better quality of right donor kidney during transplantation.

## INTRODUCTION

Live donor nephrectomy is a unique procedure in which a healthy individual undergoes a major surgery with essentially no therapeutic benefit for the donor. This special circumstance exposes the surgery to unusual and specific challenges: the benefit goes to the recipient and on the other hand the principle of “*premium non-nocere*” remains of utmost importance for the surgeon. Based on this particular situation, the donor nephrectomy should be associated with the lowest possible risks and morbidity and, simultaneously, allow the donor a speedy recovery and return to normal activities.

Laparoscopic live donor nephrectomy has emerged as an alternative to traditional extraperitoneal open nephrectomy, with the potential advantages of decreased post-operative pain, shorter hospital stay, rapid return to normal activities and clearly improved cosmesis. This procedure was originally employed with the goal of expanding the living kidney donor pool by making the procedure more appealing and acceptable [1].

**Table 1:** Technical description of mini-incision laparoscopic instrument-assisted living donor nephrectomy

**Stage**	**Procedure**
Pre-surgery	Hydrate donor with crystalloid IV fluid. Patient may receive between 4–5 L of crystalloids throughout the procedure.
	Induce general anesthesia; place a Foley catheter
	Position patient in semi-decubitus left- or right-up nephrolitomy position with 30-degree angled difference and a horizon where patient is in more supine using a maximally flexed operative table.
	Place gel pad to stabilize position and to fully raise the kidney
	Administer prophylactic antibiotic [single-dose of cefazolin (1 g)] prior to incision. Prophylactic measures of thromboembolic events include: TED and SCD’s in addition to 5,000 units of subcutaneous heparin
Surgery	Make transverse incision (7–9 cm), beginning from tip of 11^th^ rib and proceeding towards midline.
	Form superior and inferior flaps
	Muscle splitting: Split *external oblique* using 11^th^ rib as landmark (excise limited amount of cartilaginous material from 11^th^ rib to create clear plane for further muscle splitting) Split *internal oblique* muscle in the opposite direction of the muscle fibers of the *external oblique*, Divide a small amount of the lateral fibers Split *transversus abdominis* muscle
	Dissect pre-peritoneal fat and peritoneal membrane from abdominal wall in a posterior fashion using blunt dissection, followed by superior and inferior creation of space using finger dissection.
	Place combined Thompson and Omni retractors for optimal exposure
	Identify *Gerota’s fascia* and form a longitudinal, posterior opening
	Prior to handling the donor kidney administer 12.5 g of IV mannitol ≥ 3 L of crystalloid
	Dissect perinephric fat from the renal capsule, moving in order: superior to posterior to inferior
	Place bent right-angle Omni retractor to separate adrenal gland from upper pole of kidney
	Apply 2 side-to-side Sweetheart retractors medially to create optimal exposure for hilar dissection
	Using the camera of the laparoscope through a rubber band wrapped around the upper Sweetheart retractor and, in occasional cases with very high hilar lymphatics, using a harmonic scalpel, proceed to ligate and divide the gonadal vein, dissect, ligate and divide the adrenal vein in left-sided nephrectomies using endo-loop ligature.
	Ligate lumbar veins and divide followed by dissection of renal vein 1–2 cm medial to adrenal vein.
	Dissect renal artery
	Administer mannitol with 10 mg furosemide prior to mobilization of ureter.
	Dissect ureter up to the level of the iliac vessels with transection of the ureter; secure distal ureter with an end-loop ligature. Completion of the renal mobilization is accomplished by dividing all posterior and perihilar structures.
	Apply right-angled vascular clamp over the renal artery just after its origin from the aorta
	Transect renal artery using side-biting scissors
	Apply 2 consecutive endo-loops to secure the renal stump. Place double-curved C-clamp over the renal vein as medial as possible.
	Transect renal vein with side-biting scissors
	Retrieve the kidney
	Apply routine back-table handling to donor kidney; immediately transfer to recipient
	Initiate donor closing procedure Apply 4-0 Proline suture over the renal vein stump as figure-8 stay sutures to secure the renal vein from incidental retraction. Place 2 consecutive end-loop ligatures over the renal vein stump Reinforce renal vein stump with 4-0 Proline closure Reinforce renal artery with a 5-0 Proline closure
	Check for complete hemostasis
	Check for any lymphatic leaks, incidental peritoneal or pleural holes
	Remove retractors
	Apply routine procedures for closure of the muscles
	Place 2 catheters over muscles and under the skin flaps through separate exit sites for post-operative pain reduction with continuous bupivacaine hydrochloride infiltration using a pain pump.
	Close subcutaneous fat and Scarpa’s fascia with 3-0 Vicryl
	Close skin subcuticular 4-0 monocryl suture material
Recovery	Transfer patient to recovery room; stabilize before transferring to regular floor
	Remove Foley catheter on day 1; encourage patient to ambulate, use incentive spirometer

A recent United Network for Organ Sharing (UNOS) survey indicated that there is a low-risk with endoscopic techniques but greater than that associated with open donor nephrectomy techniques [2]. Inherent risks, although minimal with different laparoscopic approaches include emphysema, pneumomediastinum, pneumothorax, gas embolism, trocar inguinis, malfunctioning of endoscopic instruments, impaired handling of major complications (*e.g.*, bleeding), hidden late complications such as post-operative bleeding and cautery-induced bowel injuries. To minimize these potential risks, modifications from the original technique by Ratner, *et al*, [1] were introduced. These modifications have included both better instruments and new operative techniques such as introduction of hand-assisted laparoscopic nephrectomy [3], retroperitoneal approaches [4], and finally, hand-assisted retroperitoneal techniques [5]. These modifications and ever expanding trends of laparoscopic techniques have stimulated different centers to dramatically modify their open techniques with greater emphasis on reduction of morbidity with open operations [6-9]. However, other problems of laparoscopic donor nephrectomy are steep learning curve and higher risk of early functional impairment of the transplanted kidney due to the reduction in kidney blood flow caused by increased abdominal pressure by pneumoperitoneum [10]. Other problems include a short renal pedicle [11], higher incidence of ureteral complications, increased complications on right-sided donor due to short stump [12] and increased medical cost due to use of disposable equipment. 

Considering our strong belief in the safety of the living kidney donor, we tried to avoid inherent risks of laparoscopic techniques and instead created a minimally invasive muscle splitting anterior approach for donor nephrectomy. In this technique, we utilize advantages of open and laparoscopic approaches while simultaneously avoid inherent risks and disadvantages of both techniques. This idea was the basis for our hybrid technique of donor nephrectomy, the so-called “mini-incision laparoscopic instrument-assisted (MILIA) live-donor nephrectomy.” The objective of this report was to describe the MILIA technique and our experience with the MILIA approach to donor nephrectomy.

## MATERIAL AND METHODS

From October 2000 through February 2006, the MILIA live donor nephrectomy technique has been used in 119 cases. Early in the initial phases of the MILIA procedure in 2000, data of donors from the same center performed by partner transplant surgeons using the open-flank donor approach were collected for comparative purposes. The surgical technique is described in Table 1. This cohort of 119 cases performed by MILIA was compared to a series of 38 cases using the traditional ODN approach. Data were collected from both groups included body mass index (BMI), age, gender, ethnicity, estimated blood loss, number of units of blood transfused, intra- and post-operative complications, incision length, laterality of the kidney procured, operative time and length of stay in the hospital. Patients were matched according to BMI, age, gender and ethnicity.

**Figure 1:**
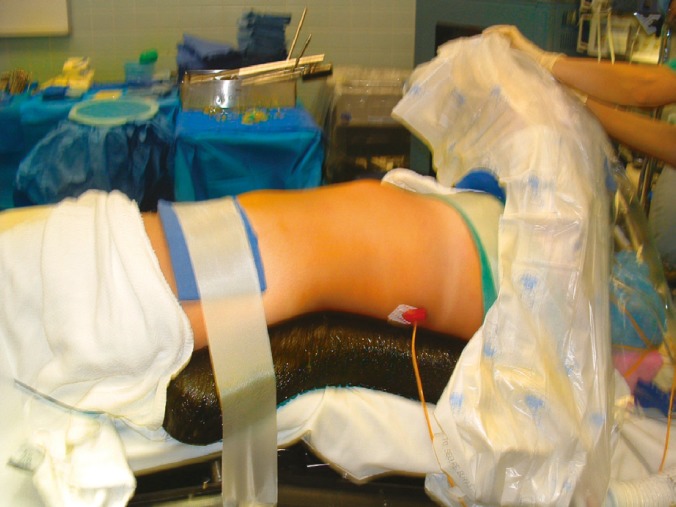
Donor positioned in maximally extended supine and semi-decubitus position, right up nephrolithotomy at 30 degree angle

**Figure 2:**
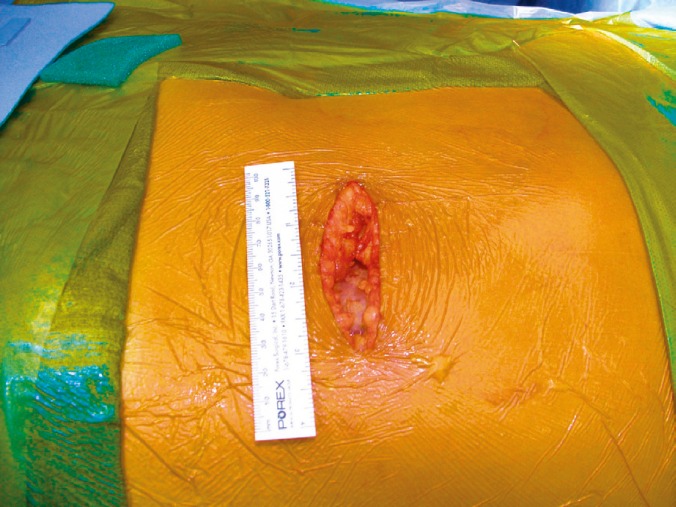
Transverse 7–9 cm incision from tip of the 11th rib towards the midline

**Figure 3:**
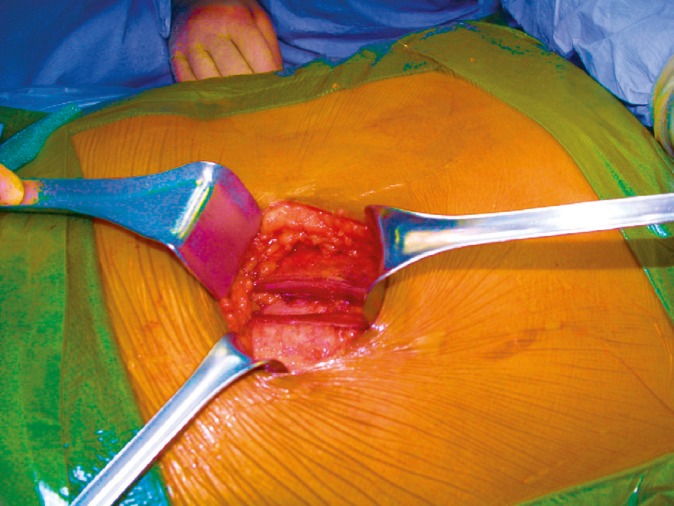
Splitting of external and internal oblique muscles

**Figure 4:**
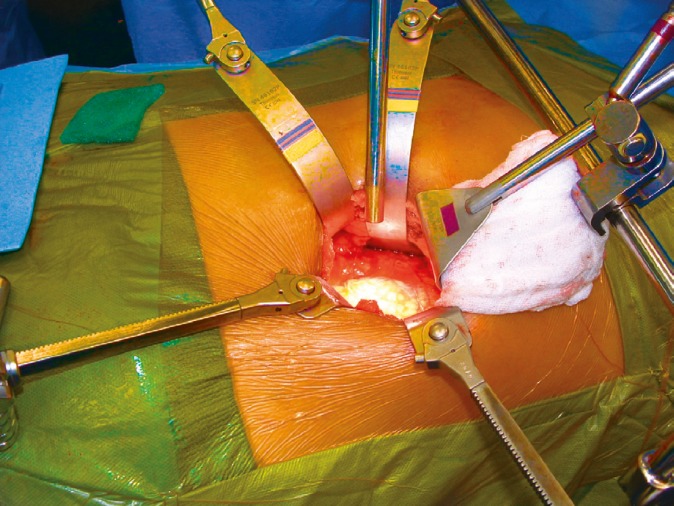
Placement of Thompson and Omni retractors and using laparoscopic camera as a light source and monitor view for the assistant surgeon

**Figure 5:**
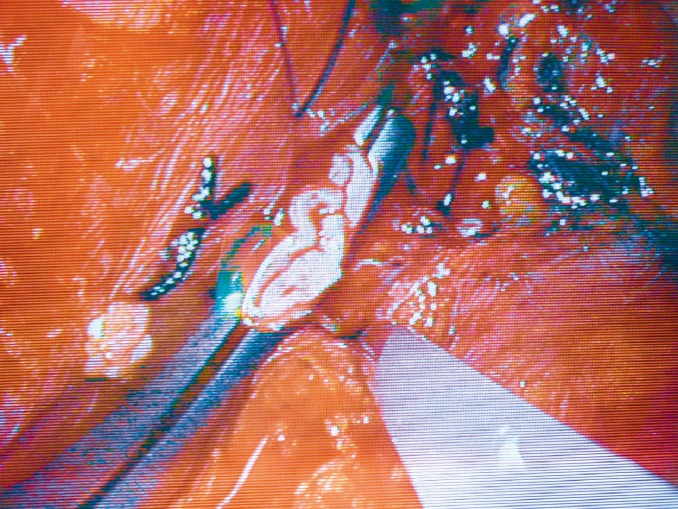
Laparoscopic view of renal fossa after kidney retrieval showing clamped renal vein during the application of endo-loop ligature

## RESULTS

The mean BMI was 27 kg/m^2^. The mean age of participants was 35 (range: 19–60) years. A majority of donors were female (58%) and Caucasian (57%). The mean BMI of the ODN group was 28 kg/m^2^ and the mean age was 35 (range: 18 –57) years.

MILIA donors experienced a significantly (p<0.0001) longer mean±SD operative time (234±47 *vs*. 197±33 min, a significantly (p<0.0001) shorter hospital stay (4±1 *vs*. 6±3 days) and less intra-operative blood loss (215±180 *vs*. 331±397 mL, p<0.02) than the ODN group. No difference occurred in the mean±SD number of units of blood transfused (0.13±0.6 *vs*. 0.34±1.0 units, p=0.13). Right kidneys were almost equally harvested in both groups (29% of MILIA donors *vs*. 26% of ODN donors). The incision length was significantly (p<0.0001) shorter in the MILIA group (8±1 cm) than the ODN group (12±3 cm). For MILIA procedures, 85% of cases experienced no intra-operative complications, while only 58% of ODN cases were without intra-operative complications (p<0.02). No difference was found in the reported post-operative complications: 85% of MILIA cases were without complications post-operatively compared to 79% of ODN cases, p=0.12). Post-operatively, MILIA donors had lower mean pain scores at one week and one month after the surgery (p<0.001). They showed significantly better post-operative recovery: earlier discontinuation of pain medications and restoration of other pre-operative activities. Moreover, they were better satisfied with their scar appearance. Scores on the short form-36 quality of life questionnaire were comparable for both groups.

## DISCUSSION

Laparoscopic living donor nephrectomy with different modifications is now the most commonly used procedure in the living donor kidney transplant process. The ideal live donor operation should have no mortality or morbidity and harvest a kidney with the best function. This will not be achieved in all cases, but with higher expectation and standards, the surgical team should take meticulous care of all important and relevant proven details in this process to get closer to the ideal result. 

Implicating the available empirical data, two techniques seem to be the best options: for endoscopy, the hand-assisted retroperitoneal approach (13); and for the open procedures, the anterior retroperitoneal approach [14, 15]. Koon Ho Rha, *et al*., have described a similar hybrid technique that we have utilized at our center [16]. In a more recent report by Hakim, *et al.*, they have also reported a fast and safe mini-incision finger-assisted nephrectomy technique applied on 225 patients [17]. However, we have been able to utilize a mixed (hybrid) technique with the goal of achieving the least-possible risk for the donor using the advantages of both extraperitoneal open and laparoscopic techniques and avoiding the disadvantages of pure intraperitoneal laparoscopic techniques or traditional open-flank nephrectomy. In the above-mentioned MILIA procedure, special attention to details of each step is of utmost importance and requires a reasonable learning curve period for training fellows. However, we believe that this curve is definitely less steep compared to the demanding laparoscopic modifications of donor nephrectomies. The endoscopic technique of a retroperitoneal approach carries the advantage of retrieving the kidney from a lower midline or Pfannestiel incision and the open technique is safer in cases of massive bleeding with no need for conversion of laparoscopic incisions to a large laparotomy incision. We believe that a retroperitoneal approach avoids potential adhesive intestinal obstructions and delayed bowel injuries and is more cost-effective without employing the expensive consumables such as hand assisted devices and endoscopic vascular staples. Finally, it should be mentioned that individual surgeons will need to determine which technique suits their skills and operative styles considering the main goal of the most important of medical principles, “first do no harm.”

Although currently we are performing the majority of our living donor nephrectomies with hand assisted laparoscopic approach started since April 2006, we still believe that the muscle splitting hybrid technique could be used in centers with limited advanced laparoscopic surgical experience and also could be considered for right donor nephrectomies in selected cases.

## CONCLUSIONS

MILIA is a viable option as an alternative for pure laparoscopic donor nephrectomy. MILIA appears to be as safe as open donor nephrectomy and may provide advantages over ODN, such as smaller incision, shorter hospital stay, and less incisional pain. Patient recovery and satisfaction after MILIA are excellent. This technique avoids the possibility of adhesive intestinal obstruction and also improves handling of major complications (*e.g.*, bleeding) of laparoscopic donor nephrectomy. Utilization of this hybrid technique is particularly feasible on smaller (BMI <24 kg/m^2^) and medium-sized (BMI <28 kg/m^2^) donors. This procedure is especially useful in right-sided donor nephrectomies because we can obtain a small rim of caval patch with the right renal vein to maximize the length the right renal vein without narrowing and compromising the diameter of the inferior vena cava.
